# Physical Constraints and Forces Involved in Phagocytosis

**DOI:** 10.3389/fimmu.2020.01097

**Published:** 2020-06-12

**Authors:** Valentin Jaumouillé, Clare M. Waterman

**Affiliations:** Cell and Developmental Biology Center, National Heart Lung and Blood Institute, National Institutes of Health, Bethesda, MD, United States

**Keywords:** phagocytosis, cell mechanics, actin dynamics, membrane, Fc receptors, integrins, receptor diffusion

## Abstract

Phagocytosis is a specialized process that enables cellular ingestion and clearance of microbes, dead cells and tissue debris that are too large for other endocytic routes. As such, it is an essential component of tissue homeostasis and the innate immune response, and also provides a link to the adaptive immune response. However, ingestion of large particulate materials represents a monumental task for phagocytic cells. It requires profound reorganization of the cell morphology around the target in a controlled manner, which is limited by biophysical constraints. Experimental and theoretical studies have identified critical aspects associated with the interconnected biophysical properties of the receptors, the membrane, and the actin cytoskeleton that can determine the success of large particle internalization. In this review, we will discuss the major physical constraints involved in the formation of a phagosome. Focusing on two of the most-studied types of phagocytic receptors, the Fcγ receptors and the complement receptor 3 (αMβ2 integrin), we will describe the complex molecular mechanisms employed by phagocytes to overcome these physical constraints.

## Introduction

Internalization of large particulate material by phagocytosis is a fundamental and well-conserved cellular mechanism of eukaryotic organisms. It enables multiple essential functions from unicellular organisms to arthropods to mammals: uptake of microbes as nutrients by single cell organisms like amoebae, removal of dead cells during tissue development or cell turnover, and clearance of microbes as a first line of defense against infection (1). Seminal work by Korn and Weisman showed that amoeba ingested multiple small particles together within the same vacuole through macropinocytosis, whereas larger particles ≥0.5 μm were phagocytosed individually and appeared tightly surrounded by a membrane derived from the plasma membrane (2). In addition, like macropinocytosis, phagocytosis is characterized by its reliance on the actin cytoskeleton, as inhibition of actin polymerization drastically reduces internalization of large particles (3–5).

While most cells can endocytose small molecules or molecular complexes, the capacity to phagocytose larger particles is not equally shared. In mammals, phagocytosis of micron-sized microbes is the prerogative of specialized innate immune cells, namely neutrophils, macrophages, monocytes and dendritic cells, also often referred as “professional phagocytes.” Physical characteristics of the particulate material, such as its shape, size and mechanical properties vary for each target and affect the success of internalization (6–9). However, the versatility and engulfment capacity of professional phagocytes is remarkable. For instance, a professional phagocyte can engulf particles substantially larger than themselves, such as IgG-coated microspheres up to 20 μm in diameter for bone-marrow derived macrophages that measure about 14 μm in suspension, or 11 μm IgG-coated microspheres for 4 μm human neutrophils (10, 11). How can they achieve such a feat?

## General Principles of Internalization by Phagocytosis

### Internalization of Large Particles Through Zipper and Trigger Mechanisms

**Figure 1:**
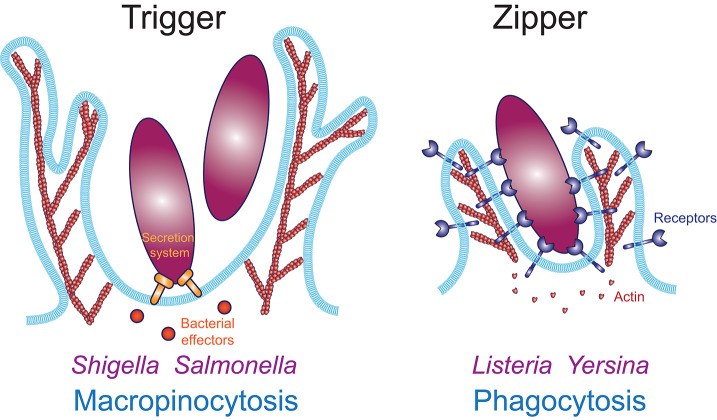
Actin-based internalization mechanisms of large particulate materials. The trigger mechanism **(Left)** enables internalization in an adhesion-independent manner. Macropinocytosis is a trigger mechanism typically induced by growth factors, such as MCSF or EGF. Bacterial pathogens like *Shigella* and *Salmonella* induce their internalization via a trigger mechanism using a type III secretion system to inject effectors inside the host cell, which induce actin polymerization to induce local ruffle formation which surround and engulf the bacteria. Numerous viruses also enter their host cell through macropinocytosis. The zipper mechanism **(Right)** requires adhesion to host cell receptors along the entire surface of the particle. Converging evidence demonstrates that phagocytosis occurs through a zipper mechanism.

Two fundamentally distinct mechanisms have been proposed for the internalization of large particulate material: the trigger mechanism where discreet signaling elicits formation of actin-based plasma membrane protrusions that non-specifically surround nearby material, and the zipper mechanism where sequential engagement of cell surface receptors to ligands on the target particle leads to a complete wrapping of the particle by the plasma membrane (Figure 1). The trigger mechanism is typified by intracellular pathogens like *Shigella* and *Salmonella*, which induce their uptake into phagocytes and non-phagocytic cells by injecting effectors via a syringe-like type III secretion system, without relying on adhesion to a specific receptor (12). Those effectors hijack the host cell signaling and actin polymerization machinery to trigger the formation of large ruffles that lead to the internalization of the bacteria in a mechanism that resembles macropinocytosis (13, 14). This was demonstrated by Galán et al., who showed that internalization of a non-invasive strain into epithelial cells could be triggered by the addition of wild type *Salmonella* (15). In contrast, other pathogens like *Yersinia* and *Listeria* employ a zipper mechanism to invade non-phagocytic cells, which requires binding of each invasive bacterium to the host cell receptors β1 integrins and E-cadherin, respectively (12, 16, 17). This illustrates that micron-sized particles like bacteria can be internalized by mechanistically distinct processes defined as trigger and zipper mechanisms. Because the trigger mechanism is limited to a very small number of specific examples, this review will focus on the zipper mechanism which has been demonstrated to mediate phagocytosis across multiple cell and receptor types and for a wide range of target particles.

### Evidence Supporting the Zipper Mechanism for Phagocytosis

A series of foundational studies from Samuel Silverstein's lab demonstrated that phagocytosis occurs through a zipper mechanism (18–20). In a first study, macrophages were exposed to red blood cells (RBC) coated with F(ab')_2_ fragments, which do not bind FcγRs and were not internalized. When IgG-opsonized bacteria were added, those were internalized, while the F(ab')_2_-coated RBCs remained on the surface, demonstrating that internalization of RBCs could not be induced by another uptake event, ruling out the trigger model. In contrast, addition of an IgG that bound the F(ab')_2_ fragments, providing a ligand for FcγRs, led to the internalization of the RBCs, demonstrating that particle internalization required direct surface receptor engagement, in agreement with the zipper model (18). Next, IgG or complement-opsonized RBCs were added to macrophages in conditions allowing binding but preventing internalization. Upon switching to permissive conditions, phagocytosis was prevented if receptors were blocked or if the opsonins were removed on the unengaged surface of the particle (19). This suggested a requirement for circumferential engagement of receptors, which was further demonstrated using lymphocytes coated with IgGs, either uniformly or on only one arc of their circumference. Remarkably, the latter were not internalized unless another IgG that bound their entire surface was added (20). These experiments demonstrated that the initial engagement of phagocytic receptors was not sufficient for particle internalization, but further recruitment of receptors was required to sequentially engage the entire surface of the particle, like a zipper, to drive engulfment. These results were confirmed more recently with asymmetrically IgG-coated “Janus” particles, which were internalized with a lower efficiency than particles evenly coated with the same amount of IgG (21). Contrary to a trigger mechanism, where particles can be captured by ruffles without direct surface-to-surface binding, the zipper model implies a very close interaction between the particle and the phagocyte surface. Experiments using a frustrated phagocytosis model demonstrated that the surface of contact with the macrophage was so tight it excluded molecules as small as 50 kDa (22). Together, these studies demonstrated that phagocytosis occurs through a zipper mechanism, which requires receptor recruitment to tightly engage the entire surface of the target particle.

Given the evidence supporting the zipper model, we will focus on the essential physical constraints associated with the uptake of large particulate material through a zipper mechanism, and the molecular mechanisms employed by professional phagocytes to overcome these constraints. Detailed discussions of the molecular mechanisms underpinning the trigger model can be found in recent reviews (23, 24). In addition, recognition of the surface molecules of phagocytic targets involves a plethora of receptors, which elicit distinct signaling pathways, which have been reviewed elsewhere (25–27). Here we will focus on the mechanisms described for two of the best-studied pathways in mammalian professional phagocytes: Fc-mediated phagocytosis, which involves binding of Immunoglobulin g (IgG) to Fc γ receptors (FcγR), and complement-mediated phagocytosis, which involves binding of the complement molecule iC3b to αMβ2 or αXβ2 integrins, also named complement receptors (CR) 3 and 4, respectively.

## Overview: Physical Orchestration of Phagocytosis

**Figure 2:**
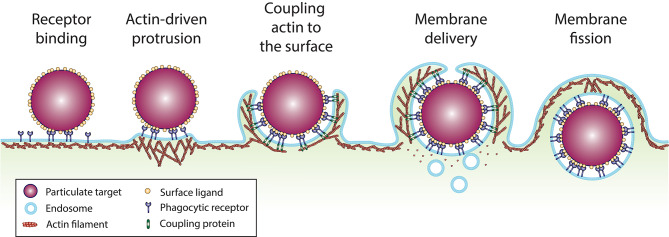
Sequence of events involved in particle uptake by phagocytosis. (1) Phagocytic receptors (dark blue) diffuse along the plane of the plasma membrane and bind ligands (yellow) at the surface of the particulate target (purple). (2) Ligand binding and clustering of the receptors elicits signaling that activates actin polymerization, which generate a protrusive force against the plasma membrane. (3) Anchorage of the actin cytoskeleton to ligand-bound receptors on the particle surface by coupling or tethering proteins, tangentially to the direction of actin polymerization, enables membrane protrusions to extend over the particle surface. (4) For large particulate targets, mobilization of membrane reservoirs from surface folds of the plasma membrane and intracellular vesicles provides the required membrane surface area to envelop the particle. (5) Once the particle is fully enveloped and the protrusions reach a meeting point, membrane fission enables the separation of the phagosome from the plasma membrane.

Uptake of large particles represents a physical challenge for the cell. However, while numerous physical constraints could be proposed intuitively, mathematical modeling combined with biophysical measurements and quantitative imaging has helped decipher which physical constraints are likely to be the most critical for phagocytosis. In the following part of this review, we will focus on five physical constraints that appear to be decisive for phagocytosis: (1) cell-surface receptors binding to ligands on the target particle, (2) generation of a protrusive force to overcome cortical tension to initiate phagocytic cup formation, (3) tangential coupling of the protrusion along the particle surface to advance the phagocytic cup, (4) membrane surface area availability, and (5) membrane fission to close the phagosome and internalize the target particle (Figure 2).

## Receptor Binding: Role of Receptor Affinity, Diffusion, and Accessibility

Receptor binding is the first essential aspect of the zipper model. However, it is determined by several parameters: the affinity of the receptors for the ligands, the lateral diffusion of the receptors in the plasma membrane, and the accessibility of the ligands. These parameters are not necessarily fixed and can be dynamically regulated by complex molecular mechanisms.

### Receptor Properties Are Essential Determinants of the Zipper Mechanism

Binding to receptors is imperative for internalization by a zipper mechanism. The dependence of receptor binding on receptor affinity, diffusion and ligand density has been formalized in mathematical modeling (28, 29). In addition, one model suggests that in the absence of actin polymerization to drive protrusion of the phagocytic cup, a passive zipper based on receptor diffusion and random membrane fluctuations could be sufficient to mediate internalization of small particles (30). In that model, internalization is slow, with highly variable phagocytic cups, and requires a low surface tension. Consistent with this model, inhibition of actin polymerization by cytochalasin D does not prevent internalization in 60 min of small IgG-opsonized beads by FcγR-transfected fibroblasts or bone-marrow derived macrophages (BMDM) (30, 31). This suggests that receptor affinity and diffusion are critical for phagocytosis, and in certain circumstances are sufficient to drive internalization.

### Conformational Changes Can Regulate Receptor Affinity

**Figure 3:**
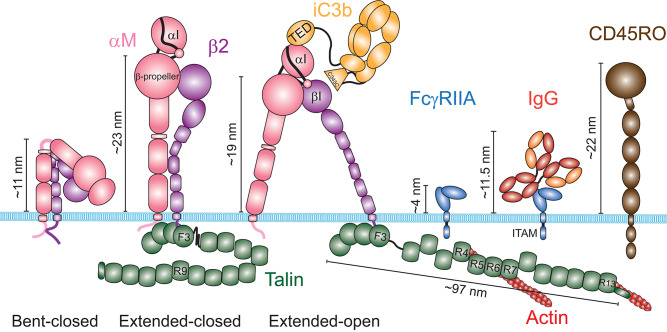
Schematic of the structures of the integrin αMβ2, the Fcγ Receptor IIA and the phosphatase CD45. The integrin αMβ2 (left) exists in at least three distinct conformations: a bent-closed conformation associated with a low affinity for its ligands, an extended-closed conformation associated with an intermediate affinity, and an extended-open conformation associated with a high affinity for its ligands. The current model suggests that integrins are maintained in an autoinhibited bent conformation by the interaction of the cytosolic domains of the α and the β chains. The switch from a bent to an extended conformation requires binding of Talin to the cytosolic domain of the β chain. The pulling force generated by the actin cytoskeleton through Talin induces the open conformation, when the integrin is attached to an immobile ligand. The ligand iC3b binds through its TED domain to the αI domain of αM. Some evidence suggest that the C345C domain could associate with the βI domain of β2, in addition to the TED-αI association. Contrary to integrins, the structure of FcγRIIA shows no conformational change upon binding to an immunoglobulin G. Average heights estimated from the membrane surface for these receptors in each conformation and for the RO isoform of CD45 are shown. For the extended conformations of αMβ2, the indicated height corresponds to the height of the β-propeller. Talin was not schematized to scale, but its estimated length at a focal adhesion is indicated.

The capacity of a receptor to bind a ligand at equilibrium is defined as its affinity. The FcγR and integrin-based phagocytic receptors have very different properties in terms of regulation by receptor-ligand affinity. The different isoforms of FcγRs have different affinities for the various IgG isotypes (32). However, structural studies showed that binding to FcγRs is not associated with conformational changes, suggesting that their affinity is constant (33). In contrast, α/β integrin heterodimers can switch between three major conformations, which have vastly different affinities for their ligand [Figure 3; (34)]. In the absence of stimulus, β2 integrins largely adopt a bent conformation that is associated with a low affinity for their ligand. Engagement of selectins, TLRs, cytokine receptors or immunoreceptors induces inside-out signaling, which involves activation of the GTPase Rap1. This leads to a kindlin- and talin-mediated unfolding of the heterodimer extracellular domains into an extended-closed conformation. Binding of the αI domain to an immobilized ligand enables the actin cytoskeleton to exert a pulling force on the integrin β2 chain through talin, which separates the α and β chains, and rearranges the ligand binding site and the adoption of the extended-open conformation (35, 36). For αLβ2-ICAM-1 interactions, the extended-closed conformation shows an increase of affinity of only 10-fold over the bent conformation, and the force-mediated opening of αLβ2 increases the affinity of the extended-open conformation for its ligand by over 5,000-fold (37). However, the affinity of iC3b for the various αMβ2 conformations has not been determined as precisely. Nevertheless, the application of a pulling force on the iC3b-αMβ2 bond has been shown to increase the bond lifetime, a phenomenon called catch-bond (38, 39). These observations support the model of a force-based change of the αMβ2 conformation, which drastically increases its affinity for iC3b, and thus likely has important implications for increasing phagocytic efficiency.

### Receptor Diffusion Is Dynamically Regulated by the Actin Cytoskeleton

In addition to affinity, the ability of receptors to find and bind to their ligand is dependent on their lateral diffusion within the plasma membrane (28, 29). However, super resolution microscopy suggested that FcγRs are not evenly distributed at the nanometer scale (40), implying that constraints on their distribution must exist. Indeed, single molecule tracking studies showed that the diffusion of FcγRs along the plane of the plasma membrane is not free, but is heterogeneous and restricted by the membrane-associated actin cytoskeleton (41).

Numerous studies have now shown that the cortical actin cytoskeleton locally constrains the diffusion of both proteins and lipids of the plasma membrane. Together these studies lead to the model of a diffusion constrained by a “fence” formed by the network of actin filaments in the cortex, which form “corrals” that are connected to “pickets” comprised of trans-membrane proteins linked, directly or not, to actin filaments (42). Ultra-fast single molecule tracking shows that diffusion within actin corrals is free, but movement between corrals is limited (43). According to the fence and picket model, friction against the pickets would impede the diffusion of molecules within the membrane, including lipids and proteins associated with the outer leaflet of the plasma membrane. It should be noted that while the cortical actin cytoskeleton is dynamic and constantly reorganizes, this occurs slowly compared to the diffusion of mobile membrane proteins and lipids (42, 44). CD44 is an abundant transmembrane protein, that associates with actin filaments through ezrin, and appears to play a major role as a picket in macrophages by restricting the diffusion of FcγRs (45). Interestingly, CD44 also binds hyaluronan, which forms a pericellular coat that curtails FcγR diffusion. This implies that CD44 is a major picket protein in macrophages that restricts FcγR diffusion, and is constrained by two fences, the intracellular actin cytoskeleton and the extracellular hyaluronan network.

The fence that restricts FcγR diffusion is dynamically regulated. Prior to their engagement, FcγR diffusion and clustering can be modulated by tyrosine kinase-mediated reorganization of the actin cytoskeleton, in response to environmental cues sensed through integrins, toll-like receptors (TLR) or cytokine receptors (41, 46–49). Monte Carlo simulations and experimental evidence suggest that a decrease of receptor confinement and receptor clustering affect receptor engagement, implying that environmental cues can prime phagocyte responsiveness via the organization of the cortical actin cytoskeleton (41, 45–47). This effect is rather complex however, as receptor engagement also depends on the density of the ligand at the surface of the target, which can vary greatly in physiological conditions, and the affinity of the receptors for the ligand, which depends on the IgG isotype. On the other hand, diffusion at the surface of bacteria is very limited (50, 51). During phagocytosis, as polymerization increases actin density around the cup, the diffusion of un-engaged FcγRs and even lipids become more restricted within the cup (41, 52). Thus, dynamic regulation of receptor diffusion could favor directional actin polymerization by allowing formation of new signaling clusters at the edge of the phagocytic cup where the FcγRs remain mobile, rather than within the cup where un-engaged receptors are restricted.

In contrast to FcγRs, diffusion properties of αMβ2 integrins are not firmly established. However, by analogy with studies done on αLβ2, they are expected to depend on the integrin conformation. Super resolution microscopy suggests that αLβ2 forms nanoclusters in the absence of stimulation, implying complex diffusional properties (53). Also, whereas the majority αMβ2 or αLβ2 appear to be immobile in resting cells, inside-out activation leads to a marked increase of the mobile fraction (54, 55). This is somewhat surprising since, as for other integrins, β2 inside-out activation requires binding of its cytosolic domain to talin (56–58). However, the lower mobility observed at rest could be due to binding of bent β2 integrins to ICAM-1 on the phagocyte's own membrane (59). In addition, consistent with cytoskeleton association through talin, extended-open αLβ2 integrins are relatively immobile (55). The respective contributions of the regulation of integrin affinity by conformational change or avidity by clustering has been a matter of debate. However, it is now apparent that the increase of affinity upon integrin conformational change is so large that, in the case of surface-associated ligands such as iC3b, the force-mediated switch to an extended-open conformation is predominant over diffusion and clustering for αMβ2 engagement.

### Access to the Receptors Limits Target Binding

In addition to diffusion driving a searching behavior, in order for receptors to reach their ligands on the target surface they must be accessible. However, at the surface of phagocytes, the glycocalyx forms a thick layer composed of large highly glycosylated membrane proteins, which can interfere with receptor binding. For instance, because the ectodomain of CD45RO, the main CD45 isoform expressed by macrophages and neutrophils, extends about 22 nm above the membrane, it could sterically block binding of IgG to FcγRs, which form a 11.5 nm complex [Figure 3; (60, 61)]. This size difference led to the idea that engagement of immunoreceptors would bring the two surfaces so close it would locally prevent CD45 diffusion into this site because of its larger size, excluding CD45 by “kinetic segregation” (62, 63). Consistent with this, CD45 appears to be excluded from FcγR engagement sites, depending on the length of CD45 ectodomain and the size of the antigen associated with the IgG (64, 65). The segregation of CD45 has important implications because its cytosolic domain is a phosphatase that regulates FcγR signaling. The observation that short antigens induce higher tyrosine phosphorylation of FcγRs and particle internalization than longer antigens, independent of receptor density, confirms the notion that steric constraints can regulate receptor signaling (64). These findings also suggest that the mechanism of CD45 exclusion induced by liquid-liquid phase separation of signaling clusters, as shown for the T cell receptor in a reconstituted system, might not be sufficient to segregate CD45 and FcγRs in macrophages (66). In addition, the close apposition of the two surfaces could facilitate engagement of nearby receptors by kinetic segregation, facilitating the formation of clusters, as proposed for the T cell receptors (62). Thus, CD45 and FcγRs engagement are mutually exclusive by steric constraints. Therefore, while the presence of large surface protein like CD45 can sterically preclude FcγR binding to IgG, CD45 local exclusion can promote FcγR signaling. In addition, a recent report showed in neutrophils that αMβ2 in its bent conformation can bind FcγRs *in cis*, impeding IgG access to FcγRs (67). This inhibition can be lifted by αMβ2 inside-out activation through cytokines or perhaps the engagement of FcγRs that remain available, facilitating further FcγR binding (54, 67). Thus, the occlusion of FcγRs appears to be a general mechanism that can be tuned dynamically to regulate FcγR binding.

In contrast to FcγRs, whether binding to αMβ2 enables glycocalyx exclusion on phagocytes remains to be explored. Analysis of αLβ2 height in T cells by iPALM showed that the β-propeller domain stands ≈23 nm above the membrane when β2 integrins are activated, to which the length of the αI domain and the ligand should be added (36, 68). This height of αLβ2 measured on cells is in agreement with the heights of αMβ2 and αXβ2 seen in structures obtained by electron microscopy. Although the actin cytoskeleton pulling on ligand-bound αLβ2 generates a tilt of the β chain that reduces its height by 4 nm, it still remains close to the height of CD45 (68, 69). This is in stark contrast with the much larger tilt observed for fibronectin-bound αVβ3 integrins on the surface of fibroblasts, which brings the integrin headpiece within a few nanometers from the plasma membrane (70). Consistent with this, binding of integrins to the extracellular matrix (ECM) appears to exclude the glycocalyx in breast cancer cells (71). Similarly, activation of integrins by FcγR signaling promotes the segregation of CD45 and facilitates further engagement of FcγRs (54, 65). Together, these observations suggest that the height of αVβ3 and possibly other integrins can be reduced enough to exclude CD45, while β2 integrins might remain too tall, suggesting that size-dependent kinetic segregation may not operate in the case of β2 integrin-mediated processes on leucocytes.

## Generation of Protrusions by the Actin Cytoskeleton

While internalization is possible through receptor binding solely driven by passive diffusion and random membrane fluctuations, this remains slow and highly inefficient for large particles, unless work is produced to promote cell surface deformation (30). Consistent with this notion, multiple models suggest that a protrusive force is required to deform the cell around the target particle to initiate formation of the phagocytic cup (11, 29, 72). Compelling evidence indicate that this protrusive force is generated by the actin cytoskeleton. However, to date, structural information regarding the actin organization within the cup remains limited. Thus, we will look at how the general principles involved in actin-based protrusions, largely learned from studies of cell migration, apply to phagocytosis.

### Actin-Based Protrusion Is a General Feature of Phagocytosis

Actin polymerization facilitates phagocytosis (3–5). Particle binding to FcγRs or many other receptors is associated with the formation of thin actin-filled membrane protrusions, usually called pseudopods, which extend around the targets (25, 73, 74). In contrast, early studies by electron microscopy suggested that αMβ2-mediated phagocytosis occurred by sinking of the particle into the cell body (73, 74), however, thin protrusions surrounding iC3b-opsonized particles have since been observed by electron microscopy (31, 75, 76). Moreover, three-dimensional live cell microscopy revealed the formation of actin-based membrane protrusions in all the observed phagocytic events of iC3b-opsonized particles (77). Thus, formation of actin-based protrusions that extend along the target appears to be a defining feature of phagocytosis, independent of the receptor.

### Phagosome Formation Is Driven by an Actin-Based Protrusive Force

**Figure 4:**
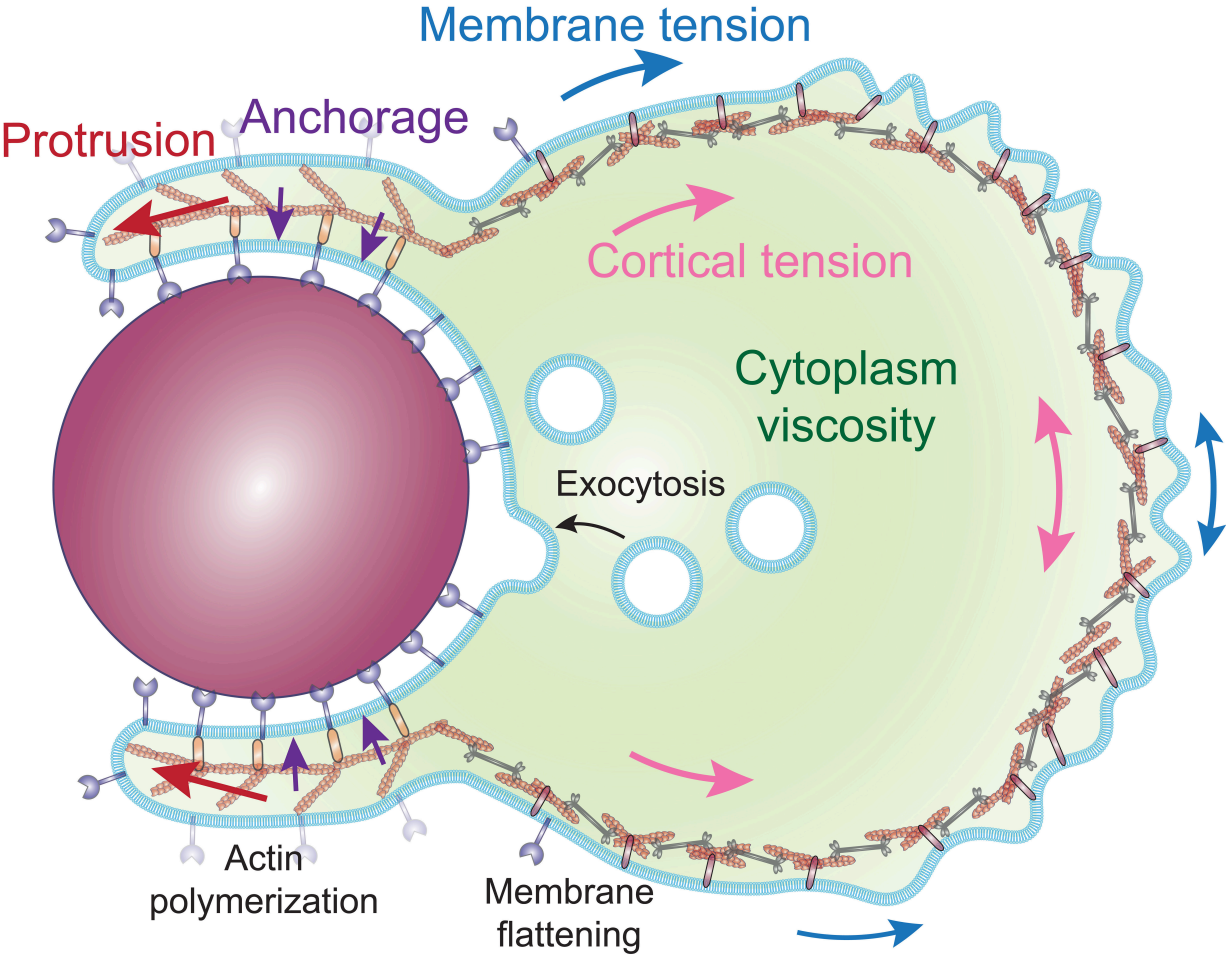
Proposed model of the cellular forces involved during internalization by phagocytosis. Phagosome formation is driven by a protrusive force (red arrows), generated by the polymerization of actin filaments, directed along the particle by an attractive force (purple arrows), and coupling proteins that anchor the actin cytoskeleton to the cell-particle interface. The protrusive force works against the surface tension, composed of the cortical tension (pink arrows) and the membrane tension (blue arrows). The rate of deformation of the cell is determined by the ratio of the surface tension and the cytoplasm viscosity, while the surface tension effectively propels the particle inward. The in-line tension of the plasma membrane is compensated by the flattening of surface folds of the membrane and the exocytosis of intracellular vesicles.

Formation of protrusions around large particles implies substantial morphological rearrangements. Modeling predicts that as the phagocytic cup grows around larger particles, deforming the cell costs more and more energy (30, 78). Internalization can be reached with a model that combines a repulsive force that pushes the leading edge forward, such as by actin polymerization, and an attractive force that anchors the cytoskeleton tangentially to the membrane engaged by the particle, which guides the protrusion around the particle [Figure 4; (11, 72)]. In contrast, a cytoskeletal expansion (gel swelling) model required unlikely parameters and failed to replicate the cup morphology observed experimentally (11).

The cortical actin cytoskeleton not only restricts receptor diffusion but the tension within the network, termed cortical tension, acts as a barrier to cell deformation. Measurements of the cortical tension during FcγR-mediated phagocytosis show that it rises when the surface area increases (from ≈ 33 to 500 pN/μm for a neutrophil engulfing a large particle) and counterbalances the protrusion of the phagocytic cup in a manner that effectively pulls the target particle inward, without requiring direct pulling of the particle by molecular motors (11, 72). Moreover, modeling predicts that the growth rate of the cup size is determined by the balance between the actin-generated protrusive force and the restoring force provided by the cortical tension, creating a bottleneck at the widest point of the particle (29). Consequently, phagocytosis can stall before the protrusion reaches the widest point of a spherical particle, but always succeeds once it passes the widest point, implying that there is no requirement for a purse-string mechanism to complete particle envelopment. In addition, the disassembly of the actin cytoskeleton observed at the base of the phagocytic cup after a few minutes of cup formation could locally reduce the cortical tension and therefore facilitate internalization (79). Finally, the energy cost for bending the membrane (≈10^−18^ J) is negligible compared to the work exerted against the cortical tension (≈10^−14^ J), consistent with the observations that cell surface tension is predominantly due to the actin-based cortical tension (78, 80, 81). These observations imply that the deformation of the cell around the target is driven by actin-generated protrusive forces, while a specific mechanism to bend the lipid bilayer such as BAR-domain containing proteins is not required. Taken together, these observations and models suggest that the major role of actin polymerization is to overcome cortical tension in order to form a protrusion around the particle, rather than pulling the particle inward.

### Comparison of the Actin Organization in Migrating Cells and at the Phagocytic Cup

**Figure 5:**
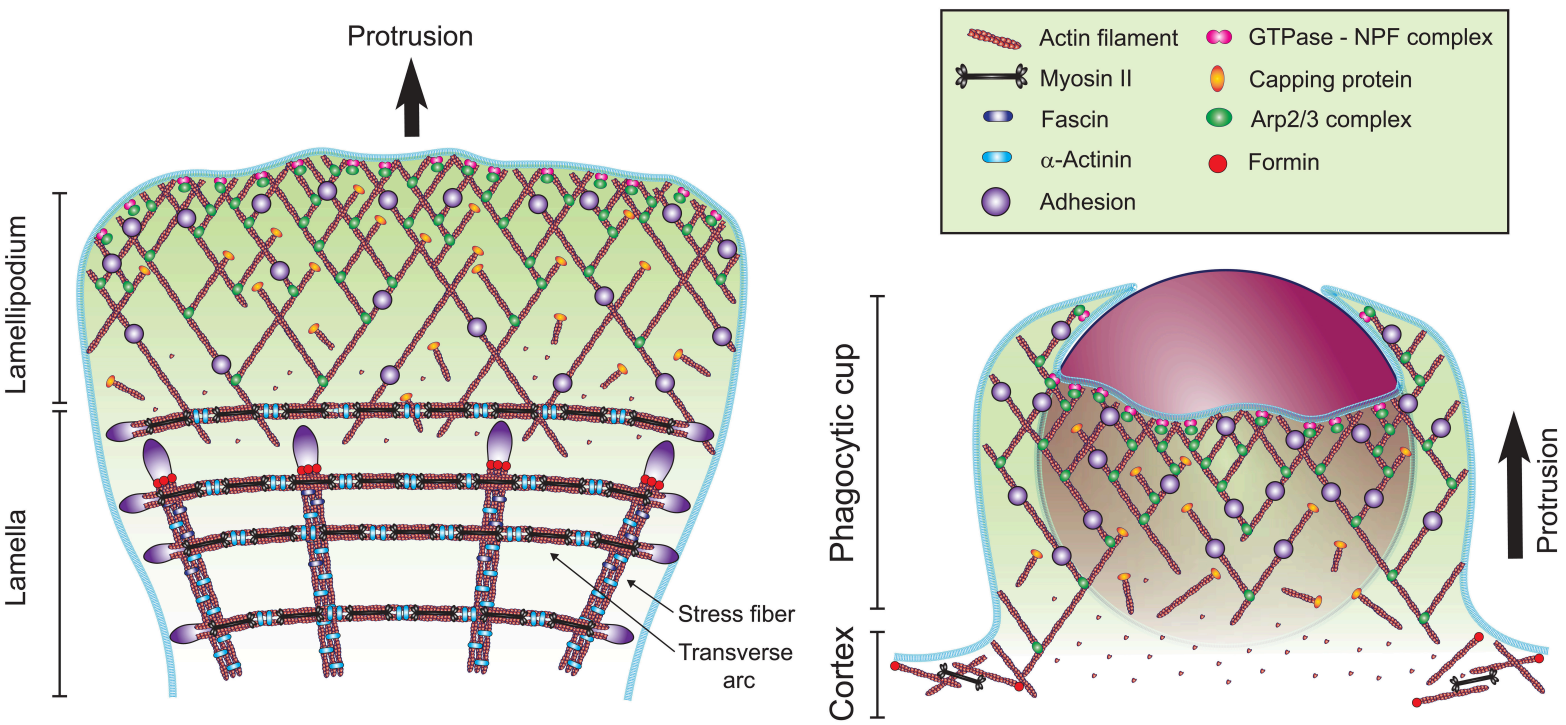
Models of the actin cytoskeleton organization at the front of a migrating cell and at the phagocytic cup. The formation of a branched network of actin filaments (red) is mediated by the Arp2/3 complex (green ellipse), which is activated at the plasma membrane by an NPF recruited by an active Rho GTPase (pink double-circle). Actin dynamics are regulated by capping proteins (orange ellipse), elongation factors, debranching, severing, and monomer binding proteins (not represented for clarity). Formation of linear actin structures, such as the stress fibers and transverse arcs at the lamella involve the bundling proteins α-Actinin (light blue rod) and Fascin (dark blue rod), actin nucleation by formins (red circle) and Myosin II mini-filaments (black dumbbell). These actin structures are stabilized on the substratum by adhesions (purple). At the phagocytic cup, adhesions are mediated by Fcγ receptors, β2 integrins or other phagocytic receptors.

The broad thin protrusions that advance over the surface of the particle during phagocytosis share many common features with the broad thin protrusions that advance over the extracellular matrix (ECM) at the leading edge of a migrating cell, which is known as the lamellipodium. The lamellipodium is composed of a branched actin network nucleated by the Arp2/3 complex and stabilized by adhesions to the ECM substrate (82–84). The lamellipodium is followed by a less dynamic and thicker region called the lamella, where actin cross-linking proteins and non-muscle myosin II motors organize the actin network into contractile bundles (85–87). Similar to lamellipodia, the Arp2/3 complex is implicated in both FcγR and αMβ2-mediated phagocytosis (31, 77, 88). Live cell SIM-TIRF microscopy of filamentous actin (F-actin) during the formation of a frustrated phagocytic cup upon engagement of αMβ2 suggests the formation of a branched actin network, with Arp2/3 localized at the leading edge, similar to a lamellipodium (77). The branched actin network of the lamellipodium generates high protrusive forces, ranging from 2 to 10 kPa in migrating cells, which would be well-suited to overcome the increasing surface tension during phagosome formation and advancement (89). Moreover, the structure of a branched network self-adapts to the load generated by membrane tension, which increases the F-actin density and resistance under higher loads (90, 91). These observation suggest that the actin-based protrusions formed during phagocytosis are similar to a lamellipodium, except that the cup shape is imposed by the geometry of the engaged particle (Figure 5).

### Regulating Factors of Actin Dynamics at the Lamellipodium and the Phagocytic Cup

How is Arp2/3 activated during phagocytosis? In lamellipodia, directed actin polymerization involves the recruitment and activation of Arp2/3 at the leading edge, which requires its interaction with the VCA (verprolin, connecting, acidic) domain of a nucleation promoting factor (NPF). In macrophages and neutrophils, the Wiskott-Aldrich syndrome protein (WASP) is an abundantly expressed NPF and is involved in FcγR-mediated phagocytosis (92–95). WASP activation requires binding to the GTP-bound Rho-family GTPase Cdc42 and to phosphatidylinositol 4,5-bisphosphate [PI(4,5)P_2_] (95, 96), which are both localized at the tip of protrusions during FcγR-mediated phagocytosis (97, 98). Cdc42 is required for FcγR-mediated phagocytosis and its recruitment is favored by the adaptor Nck (92, 99–101). The GTPases Rac1 and Rac2 are also activated during FcγR-mediated phagocytosis, though more at the base of the cup, and can activate Arp2/3 through the NPF WAVE2 (98, 102). Rac dominant negative and Rac2 silencing suggested that Rac family proteins are required for FcγR but not for αMβ2-mediated phagocytosis (100, 101). However, *rac1 rac2* double-knockout macrophages are defective in both FcγR and αMβ2-mediated phagocytosis (75) and RhoG, a Rac-related GTPase, has been implicated in both pathways (100). Thus, Arp2/3 could be activated at the edge of the phagocytic cup by various Rho family GTPases depending on the engaged receptors.

Actin assembly and motion exhibit a stereotypical organization in protrusive cellular structures. Fluorescent speckle microscopy shows that the lamellipodium is characterized by assembly at the leading edge followed by disassembly a few microns back in a process known as treadmilling (86). In addition to Arp2/3, which localizes in the first micrometer of the leading edge and has a shorter lifetime than actin, actin dynamics are strongly affected by capping proteins, which block incorporation of new monomers at the filament barbed end within ≈ 0.5 μm of the edge (103). In contrast, Ena/VASP proteins prevent capping of actin filaments and can affect polymerization by recruiting G-actin-profilin complexes and by reducing branching (104). In macrophages, knockout of the gene coding for the capping protein CapG reduces FcγR and αMβ2-mediated phagocytosis (105). Furthermore, VASP is strongly recruited at the FcγR phagocytic cup, independently of the classic Rho GTPases, and inhibition of Ena/VASP proteins impairs uptake (106). Thus, actin polymerization is regulated by the combined activities of Arp2/3, capping proteins and elongation factors that may mediate treadmilling at the phagocytic cup.

### Mechanism of Actin Depolymerization at the Phagocytic Cup

As micron-size particles are too large to pass through the mesh of a branched actin network in the cortical cytoskeleton, F-actin disassembly and clearance at the base of the phagocytic cup appears to be essential for particle internalization (79). Remarkably, actin disassembly occurs even upon forced activation of Rac at the phagosome, but coincides with and requires PI(4,5)P_2_ hydrolysis by phospholipase C, downstream of PI3K (79). This suggests that actin clearance does not simply require inhibition of Rho GTPase signaling, but active regulation of actin network disassembly. The proteins ADF (actin depolymerization factor), cofilin and gelsolin can sever actin filaments into shorter polymers and accelerate disassembly of the slow growing ends of the filaments. Yet, as severing also creates a new fast growing end, it increases the rate of filament turnover but does not necessarily lead to a reduction in F-actin concentration (107). Aip1/Wdr1 binds to cofilin and causes net depolymerization (108). In addition, the Arp2/3 complex can be inhibited directly by several proteins, including coronins and arpin (109, 110), and coronins can synergize with cofilin to sever ADP F-actin (111). Several studies have reported a role for cofilin in FcγR and αMβ2-mediated phagocytosis (112–114). As cofilin is inhibited by PI(4,5)P_2_ (115), it is likely to be inactive at the tip of the protrusions, but become activated and induce actin depolymerization as soon as PI(4,5)P_2_ is hydrolyzed at the base of the cup. On the other hand, gelsolin, which is activated by Ca^2+^, enhances FcγR but not αMβ2-mediated phagocytosis in neutrophils, and is dispensable in macrophages (105, 116). The role of coronin-1 in phagocytosis has been a matter of debate (117–120). However, arpin is recruited to the forming phagosome, reduces F-actin density and enhances uptake by FcγRs, consistent with its activity as an Arp2/3 regulator (121). Thus, actin depolymerization at the base of the cup could be promoted by cofilin, activated upon PI(4,5)P_2_ hydrolysis, in conjunction with Arp2/3 inhibition by arpin.

### Does Myosin II Play a Role in Phagosome Formation?

While non-muscle myosin II has been localized at the phagosome, its contribution to internalization remains unclear (122). Myosin IIA, the predominant isoform expressed in leukocytes, assembles into 320 nm long bipolar filaments with an average of 14 myosin heads at each end of the filament to form a contractile unit (123, 124). These bipolar filaments pull actin filaments into antiparallel bundles, such as dorsal arcs in migrating cells, or concentric arcs at the immunological synapse (125, 126). The polarity of actin at the leading edge and the directionality of myosin motors result in myosin pulling actin in the opposite direction of the leading edge protrusion to drive actin retrograde flow (86, 127, 128).

Despite this putative negative effect on protrusion formation, several studies have suggested a role of myosin II in particle uptake during FcγR and αMβ2-mediated phagocytosis (129–131), whereas in other studies, inhibition of myosin II motor activity had no effect on particle internalization (31, 77). Furthermore, while myosin II-driven actin arcs are clearly apparent by SIM-TIRF microscopy at the immunological synapse, no such actin structures are visible in αMβ2-mediated phagocytic cups (77, 126). In addition, traction stresses at αMβ2 phagocytic cups are similar to those measured upon myosin II inhibition in migrating cells (77, 132). Likewise, myosin II-dependent contractility is only observed at the FcγR phagocytic cup after the cell surface increases by over 225% (133). Thus, it seems unlikely that myosin II plays a role in the advancement of the phagocytic cup, but it could participate in other aspects of phagocytosis. Indeed, the conflicting effects of myosin II in phagocytosis might be explained by experiments that combined tracking the displacement of IgG-opsonized beads with real-time measurements of cortical tension, which suggested that particles were not directly pulled by molecular motors, but that the increase of cortical tension effectively propelled particles inward (11). As myosin II activity increases cortical tension (80), it would impede protrusion around the particle, but facilitate particle inward movement (29). The effect of myosin II inhibition might thus be variable for different phagocytes since they exhibit distinct cortical tensions (134). Interestingly, myosin II also promotes actin disassembly at the rear of migrating cells (135) and in the cytokinetic furrow of dividing cells (136, 137). Therefore, a contribution of myosin II in actin clearance at the base of the cup is worth considering.

## Coupling the Protruding Cup to the Particle Surface

The actin cytoskeleton is capable of generating appropriate forces to deform the phagocytic cell around the particulate target. However, quantitative imaging combined with modeling suggests that the actin-based pushing forces must be directed tangential to the particle surface to guide the protrusion around the particle instead of pushing it (11, 72). This implies that actin polymerization should not emanate from the phagocytic receptors, but the growing network should be anchored to the target surface tangential to the direction of the actin polymerization by molecular linkages to the phagocytic receptors or plasma membrane molecules localized at the particle interface. This concept has been previously established for mesenchymal cell migration, where experiments show that coupling of directed actin polymerization oriented tangential to the ECM surface to engaged integrins near the cell leading edge determines cell displacement along the ECM (138, 139). Furthermore, this model is consistent with the case of enteropathogenic *Escherichia coli* (EPEC), which employs a type III secretion system to inject its own receptor, Tir (140). Tir activates host cell actin polymerization perpendicular to the bacterium-cell interface, which does not result in phagocytosis, but instead leads to the formation of a broad protrusion called a pedestal that elevates the bacterium and makes it surf along the host cell surface (141). This illustrates that the directionality of actin polymerization relative to the target is critical to achieve internalization. Here we will discuss the molecular mechanism that could mediate actin cytoskeleton coupling to the particle surface during phagocytosis.

### The Molecular Clutch Model in Cell Migration

**Figure 6:**
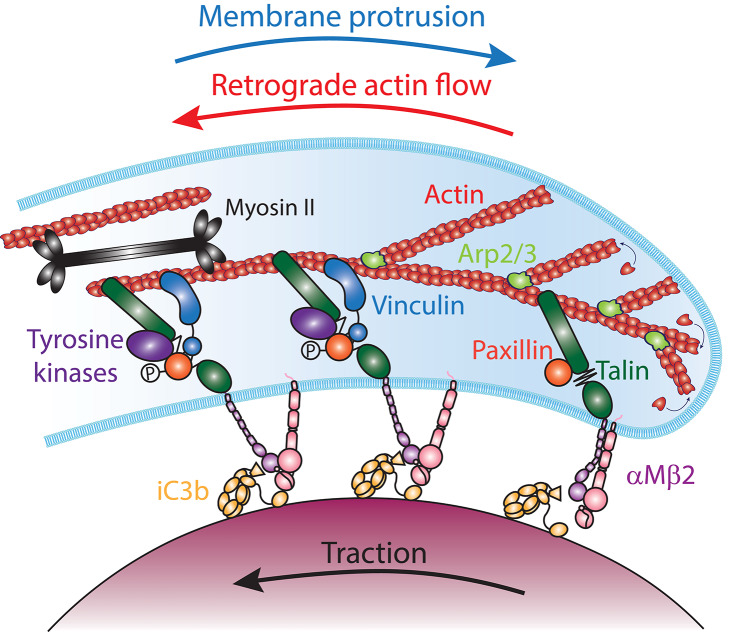
The molecular clutch model in αMβ2-mediated phagocytosis. The Arp2/3 complex (light green) nucleates actin (red) polymerization at the leading edge of the phagocytic cup. The addition of new monomers to the filaments at the membrane generates forces that push against the plasma membrane, leading to an equilibrium between membrane protrusion and the retrograde flow of actin filaments. Coupling of iC3b (yellow) -bound αMβ2 integrins (purple and pink) to actin filaments by Talin (dark green), transmits mechanical tension that switches the integrins into an extended-open conformation and provides traction on the particle. Stretching of Talin reveal Vinculin (blue) binding sites and the phosphorylation of Paxillin (orange) by tyrosine kinases (purples). This leads to the recruitment of Vinculin, which reinforces the molecular clutch to prevent its slippage, reducing the retrograde flow of actin and increasing traction and forward protrusion. In addition, contractility mediated by Myosin II (black) could increase tension across Talin and increase the actin retrograde flow and traction onto the particle, while reducing the protrusion of the phagocytic cup edge.

Because protrusion of the phagocytic cup is analogous to the lamellipodium and utilizes a similar machinery, we can extend the analogy with cell migration to learn about the mechanism of coupling the protruding phagocytic cup to the particle surface. In mesenchymal cell migration, coupling of the actin cytoskeleton to the ECM substrate is mediated by an integrin- and talin-based “molecular clutch.” At the leading edge of the lamellipodium, directional incorporation of actin monomers into the actin network generates a pushing force against the plasma membrane. The plasma membrane provides a resistive force that is large enough that unconstrained actin assembly cannot deform it, and instead the force of actin assembly results in pushing the entire actin network back from the membrane in a process termed retrograde actin flow (138, 142). To instead utilize the pushing force of actin polymerization to drive forward protrusion of the plasma membrane, a resistance force must anchor the actin network to the substrate to prevent it from sliding back. Thus, actin assembly could either drive retrograde flow or forward protrusion, depending on whether the actin is anchored to the substrate or not. Consistent with this notion, the forward movement of the leading edge is inversely proportional to the F-actin retrograde flow rate in lamellipodia of migrating cells (138, 143). Based on these observations, Mitchison and Kirschner proposed that a “molecular clutch” connects the retrograde moving actin cytoskeleton to ECM-bound trans-membrane receptors in order to propel the cell forward [Figure 6; (144)]. This molecular clutch is composed of focal adhesion (FA) proteins, which transmit actin-generated forces to integrin cytoplasmic tails, creating traction stresses onto the to ECM-bound integrin in the same direction as the retrograde flow (132, 139). While several proteins can bind both integrin tails and actin filaments directly, talin is required for cell spreading and lamellipodium stabilization (145). Thus, talin is a molecular clutch protein that couple integrins to the actin cytoskeleton in the lamellipodium of migrating cells.

Vinculin is an important talin binding partner that is thought to regulate the strength of the molecular clutch. Although talin is sufficient to link ligand-bound integrins to the actin cytoskeleton, it is a weak and labile bond (146), and vinculin is thought to reinforce the talin-actin linkage. Indeed, the linkage of ligand-bound integrins to actin retrograde flow generates tension across talin that stretches the molecule, revealing binding sites for the recruitment of vinculin in a force-dependent manner (147–149). As vinculin binds to talin and actin filaments, it reduces slippage of the molecular clutch, slowing down the F-actin retrograde flow and increasing traction onto the ECM (150, 151). Vinculin recruitment can also occur in a force-independent manner when vinculin has an open conformation, which can be promoted by its phosphorylation, or when the adaptor protein paxillin is tyrosine phosphorylated by Focal Adhesion Kinase (FAK) (152–154). Mechanical loading also induces the maturation of small nascent adhesions into larger and stronger vinculin-, zyxin-, and tyrosine phosphorylation-rich FAs, in a RhoA and mDia1 dependent manner, while ROCK and myosin II motor activity are dispensable (155, 156). However, myosin II can also stimulate FA maturation by promoting actin filament bundling (155). Thus, vinculin can be recruited to reinforce the talin molecular clutch in myosin II dependent and independent manners.

### A Molecular Clutch Is Involved in αMβ2-Mediated Phagocytosis

Although the notion of a talin-mediated molecular clutch driving cell migration is well-accepted, what is the evidence that coupling of the actin cytoskeleton to ligand-bound receptors by a molecular clutch promotes phagosome formation? Similar to integrin-mediated migration, talin is required for αMβ2-mediated phagocytosis (56, 57). Moreover, whereas the rod domain of talin, which binds actin filaments, is dispensable for target particle binding, it is required for efficient internalization (57, 77). Vinculin and paxillin are also recruited to the phagosome, and vinculin recruitment is promoted by the Syk, FAK/Pyk2, and Src family tyrosine kinases (74, 77). Since αMβ2-mediated phagocytosis involves RhoA and mDia1, they might also contribute to adhesion maturation (100, 101, 157). Furthermore, traction force microscopy shows that αMβ2 integrins are mechanically coupled to the actin cytoskeleton within the phagocytic cup through talin and vinculin, generating a pulling force tangential to the target surface that drives protrusion of the phagocytic cup (77). Thus, a talin/vinculin-based molecular clutch promotes phagosome formation by coupling actin-generated forces to αMβ2 integrins engaged to iC3b on the particle surface.

### Mechanical Coupling of the Actin Cytoskeleton Enables Mechanosensing

The talin/vinculin-based molecular cutch is known to be sensitive to the mechanical loading that occurs in response to the stiffness of the integrin-engaged substrate, enabling the regulation of cellular functions, such as cell adhesion, migration and transcriptional regulation (158). During phagocytosis, a major consequence of the mechanical coupling of αMβ2 integrins to actin-generated forces is an increased protrusion speed of the phagocytic cup edge in a manner dependent on the stiffness of the target particle. Consequently, actin-αMβ2 coupling is associated with more efficient uptake of stiff IC3b-opsonized targets, whereas soft targets are poorly internalized. Interestingly, the elastic modulus of Gram-negative and Gram-positive bacteria is in the range of 20–200 MPa (159–162), which is much higher than mammalian cells, which range from 0.2 to 20 kPa (163). Moreover, cells become stiffer during apoptosis, which promotes their internalization independent of the “don't eat me” signal mediated by CD47 (164–166). This implies that the talin/vinculin molecular clutch could contribute to target discrimination for integrin-mediated phagocytosis based on their mechanical properties.

Interestingly, internalization of IgG-opsonized particles is also stiffness sensitive, implying mechano-sensitivity mediated by FcγRs (6, 166). Mechanosensing requires that a force is applied to the target, however no protein is known to couple FcγRs to the actin cytoskeleton. How does mechanosensing occur during FcγR-mediated phagocytosis? One possibility is that FcγRs are not directly connected to the actin cytoskeleton, but the friction generated by cytoskeleton-membrane contacts such as WASP-Arp2/3 interactions, while the actin network flow relative to the membrane could effectively pull on IgG-bound FcγRs (167). Such a “loose clutch” has been proposed for the sweeping of T cell receptors along with actin flow at the immunological synapse, and is consistent with the centripetal motion of FcγR clusters when IgGs are associated with a fluid surface (168, 169). Alternatively, integrins could be responsible for mechanosensing during FcγR-mediated phagocytosis. Engagement of FcγRs activates integrins, and FA proteins such as talin, vinculin, paxillin, and α-Actinin are recruited to the FcγR phagosome (74, 170). High resolution microscopy showed that upon binding to IgG, podosomes are formed several micrometers back from the edge of the phagocytic cup (171, 172). Formation of podosomes instead of FA is typically promoted by Src family kinases, which are activated by FcγRs, combined with low contractility (173–175). The role of integrins in FcγR-mediated phagocytosis had been initially discounted since silencing of talin impaired internalization of iC3b-opsonized, but not IgI-opsonized RBCs (57). However, the combination of β1 and β2 integrin blocking antibodies, or the over-expression of talin head domain, which uncouples integrins from the actin cytoskeleton, reduces FcγR-mediated uptake (65). Importantly, contrary to other integrin adhesions, podosomes exert a pushing force normal to the surface, which could cause the indentations observed during phagocytosis of soft IgG-opsonized particles (176, 177). Therefore, by generating a normal pushing force, podosomes might not contribute directly to the leading edge protrusion, but could enable stiffness sensing of IgG-opsonized particle stiffness.

### Receptor-Independent Anchorage of the Actin Cytoskeleton

The postulated need for anchorage of the cytoskeleton to drive FcγR-mediated phagocytosis could also be supported by proteins that link actin to the membrane instead of to the receptors themselves (11, 72). In particular, myosin I is a class of small monomeric motors that bind actin filaments through their head domain and membranes through the TH1 domain of their tail (178). Myosin Ig localizes within the protrusions formed along IgG-opsonized particles, whereas myosin Ie and If precede F-actin at the edge of the protrusions (179, 180). The membrane binding tail of myosin Ig is involved in facilitating internalization, consistent with a role in anchoring the cytoskeleton to the membrane (180). Myosin Ie and If however appear to stimulate F-actin turnover within the FcγR-mediated phagocytic cup (179). Finally, the actin cytoskeleton could also be anchored to the plasma membrane through ezrin-radixin-moesin (ERM)-family proteins, which can bind to plasma membrane-associated molecules including PI(4,5)P_2_, EBP50, ICAM, or CD44, as suggested by the localization of ezrin to the forming phagosome (181). Thus, in addition to a molecular clutch, membrane-actin tethering proteins could participate in cytoskeleton anchorage during phagocytosis.

## Providing Enough Membrane to Envelop the Target

In addition to the mechanical constraints involved in deforming the cell, biophysical properties of the membrane are also critical for phagocytosis. The zipper mechanism implies that the target particle becomes entirely enveloped by a membrane. However, as the plasma membrane is essentially inextensible (182), models suggest that the membrane surface area available represents an absolute limit on the internalization of large particles (78). Consistent with this, experiments comparing uptake of beads of various sizes or the extent of spreading during frustrated phagocytosis showed that macrophages reach their limit at a fixed surface area, well before all FcγRs are occupied (10). Indeed, enveloping large particles or multiple smaller particles requires substantial membrane surface, yet neutrophils and macrophages can engulf particles larger than their initial diameter (10, 11). The forming phagosome, at least initially, is derived from invaginations in the plasma membrane (2, 183). However, the plasma membrane is poorly elastic and does not expand more than 2–4% before rupturing (182). This implies that phagocytes need to mobilize extra membrane to their surfaces in order to engulf large or numerous targets.

### The Different Sources of Membrane

Two types of “membrane reservoirs” appear to act as sources for phagosome formation: folds in the plasma membrane or intracellular vesicles, which upon mobilization and fusion with the plasma membrane increase its surface area. In support of the first model, macrophages, and neutrophils present a very rough surface as they constantly form ruffles, filopodia, and other membrane protrusions or invaginations. Early observations by scanning electron microscopy revealed that the surface of macrophages becomes smoother after phagocytosis, suggesting that membrane folds have been flattened out to provide more membrane surface to the phagosome (184). In support of the second model, Hirsch and Cohn showed that neutrophils degranulate during phagocytosis and suggested that granules might fuse with the phagosome, which was later observed directly by video microscopy (185, 186). Macrophage phagocytic capacity is reduced upon artificial expansion of the surface area of lysosomes or the depolymerization of microtubules, which are required for intracellular organelle movement, suggesting that mobilization of intracellular compartments contributes to the membrane reservoir (10). Different intracellular compartments appear to contribute to the formation of the phagosome, including recycling endosomes and late endosomes, which fuse with the plasma membrane at the forming phagosome in a SNARE protein-dependent manner (187–189). On the other hand, whereas the association of ER proteins with the phagosome suggested that the ER could contribute as a membrane reservoir, multiple experiments suggest that the ER membrane does not fuse with the plasma membrane but is recruited through the interaction of STIM-1 with ORAI, leading to peri-phagosomal Ca^2+^ signaling (183, 190). Thus, membrane appears to be provided by endocytic vesicles and granules, but not by the ER during phagosome formation.

### The Role of Membrane Tension

The regulation of membrane reservoir mobilization during phagocytosis remains poorly understood but is likely to involve membrane tension. In cells, membrane tension is defined as the membrane capacity to resist deformation and results from the combination of membrane in-plane tension, membrane bending stiffness and membrane attachment to the actin cortex (191). Experiments using the frustrated phagocytosis model suggest that the mobilization of each membrane reservoir occurs sequentially (192). First the flattening of membrane folds can provide 20 to 40 % of surface area, then exocytosis at the phagocytic cup occurs once the membrane tension reaches its maximum (192). Similarly, during internalization of large particles, the membrane tension measured outside of the phagocytic cup rises from ≈30 to 45 pN. More generally, an increase of plasma membrane tension by a hypotonic shock in macrophages and other cells results in exocytosis (192–194). This suggest that the increase of membrane tension observed during phagocytosis could be the signal that induces exocytosis of membrane reservoirs. Importantly, because the membrane is an inelastic fluid, it is generally assumed that stresses (in-plane tension) equilibrate very rapidly across the entire plasma membrane (193). So, how does focal exocytosis occur at the forming phagosome? Since the membrane tension is affected by the membrane attachment to the actin cytoskeleton, the dramatic actin reorganization at the forming phagosome may in fact locally increase the membrane tension, as has been observed at the leading edge of fast migrating cells (195). The increased membrane tension, along with the aforementioned clearance of F-actin from the base of the cup, may govern the mobilization and local fusion of intracellular membrane reservoirs during phagocytosis. While the signaling pathway(s) orchestrating these events are incompletely understood, it is known to involve the phosphoinositide 3 kinase (PI3K), which is required for internalization of particle larger than 3 μm (8, 192, 196). Thus, membrane tension can govern the mobilization of intracellular membrane reservoirs during phagocytosis, through a signaling pathway that is incompletely understood.

## Membrane Fission During Phagosome Closure

Phagosome closure is the final essential step of particle internalization but arguably the least understood. Phagosome formation occurs when the edges of the advancing phagocytic cup reach a point of contact and merge, leading to the fission of the membrane that releases the phagosome from the plasma membrane. This process is one of the most difficult aspects of phagocytosis to study because it is challenging to identify fully wrapped but unclosed phagosomes (197). Membrane fission requires bringing sites of a continuous membrane to <3 nm apart to induce merging of the outer leaflet to form an hemifission neck, followed by merging of the inner leaflet to allow separation (198). Interestingly, the observation that when two macrophages try to engulf the same target they do not fuse together at the contact site, suggests that phagosome closure involves a molecular mechanism distinct from previously described cell fusion mechanisms (122). When physical constraints are minimal, several mechanisms can elicit membrane fission without energy consumption *in vitro*. However, given the membrane tension measured during phagocytosis and the physical barrier created by the extracellular domains of surface proteins, which can impede contact between lipid bilayers, phagosome closure is likely to involve an active mechanism to drive membrane fission (199). Current evidence suggests the role of two possibly synergistic mechanisms: membrane constriction by mechanochemical proteins and membrane pushing by the actin cytoskeleton.

### Mechanochemical Proteins Involved in Membrane Constriction

Dynamin is the first and best characterized protein known to induce membrane fission and is involved in various endocytosis and organelle division pathways (200, 201). While it is not a molecular motor in the classical sense, dynamin assembles into a ring-shaped polymer that has contractile properties through its enzymatically driven hydrolysis of GTP. Constriction of the ring from a 20 nm inner diameter to 3.7 nm is achieved by a GTP-dependent conformational change by twisting, while the fission event is promoted by membrane tension (202, 203). Dynamin-2 is ubiquitously expressed and is recruited to FcγR and αMβ2-mediated phagosomes concomitantly with F-actin (204, 205). More importantly, dynamin-2 has been visualized at the phagosome closure site in a TIRF-based assay, and inhibition of dynamin activity reduces internalization. Interestingly, dynamin inhibition inhibits protrusion formation, suggesting that dynamin cross-talks with actin dynamics (205). Thus, dynamin-2 might interact with actin filaments at the edges of the phagocytic cup and induce membrane fission when the edges converge into a closure site.

In addition to dynamin-2, myosin Ic is a molecular motor recruited to the phagosome at the late stage of FcγR-mediated phagocytosis (122). Interestingly, when one IgG-opsonized RBC is phagocytosed simultaneously by two macrophages, myosin Ic localizes to the meeting point of the opposing phagocytic cups, suggesting a role in phagosome closure (122). There is no clear evidence so far that myosin I family proteins could mediate constriction. However, as a membrane-actin tether, myosin I can increase membrane tension, which could facilitate dynamin-mediated membrane fission (206). Unlike myosin Ic, myosin II has not been localized to the meeting point (122). Because myosin II plays an important role in the formation of the constriction ring during cytokinesis, it has been proposed that myosin II could assist phagosome closure by a purse-string mechanism. However, it should be noted that myosin II is required to maintain cortical tension during cytokinesis through actin bundling, whereas its motor activity is dispensable for cell division in culture and *in vivo* (207). Furthermore, the 320 nm length of myosin II contractile units makes a role in membrane fission, which occurs at a much smaller scale, highly unlikely (123). Thus, myosin I proteins are the myosins that are the most likely to contribute to membrane fission during phagocytosis.

### Does Actin-Mediated Pushing Force Participate in Membrane Fission?

Actin-based protrusive forces could also facilitate membrane fission during phagosome closure. In yeast, a major role of Arp2/3-mediated actin polymerization is to support clathrin-mediated endocytosis (208). The current model suggests that actin polymerization pushes against the plasma membrane in the area of membrane bending, where WASP is localized. Myosin I and the dynamin protein Vps1 form a ring around the clathrin-coated pit, which can tether the F-actin network to the forming endosome (209, 210). This tethering could enable F-actin retrograde flow to overcome the membrane tension and turgor pressure to pull the forming endosome away from the surface, consistent with evidence that myosin I primarily contributes to endosome inward movement (211). Furthermore, pushing force generated by actin polymerization could also facilitate membrane fission during phagocytosis by bringing the lipid bilayers closer at the closure site. Interestingly, a burst of actin polymerization is often visible at the point of closure and appears to push the phagosome away from the surface during αMβ2-mediated phagocytosis (77). Furthermore, a normal stress of about 150 Pa is observed on fully wrapped, IgG-opsonized soft particles, indicating that a pushing force, presumably generated by actin polymerization, propels the particle inward (177). Taken together these observations support the idea that actin polymerization, tethered to the membrane by dynamin-2, myosin I, and/or a talin-based molecular clutch, can promote membrane fission during phagosome closure.

## Concluding Remarks

Since phagocytosis is a cellular process broadly employed across eukaryotes, it seems likely that phagocytes employ fundamentally shared molecular mechanisms to overcome the physical constraints imposed by the internalization of large particulate material by cells. The comparison of phagocytosis with other general cellular processes, such as cell migration, shape change, cell division, or endocytosis, is very helpful to understand the molecular mechanisms that are at play. However, it also highlights the fact that we still only have pieces of the puzzle, which are largely consistent with the general framework, yet it will take major effort to complete the details of the molecular mechanisms involved and to understand how they are coordinated to enable uptake. It should be noted that while we have learned a lot from studies on the canonical receptors FcγR and αMβ2 with model particles like microspheres and red blood cells, we can speculate that the general concepts exposed here will apply broadly to phagocytosis in physiological conditions, with a number of nuances and additional physical constraints.

For instance, microbes not only vary in size but can exhibit an array of diverse shapes, which present distinct physical constraints. It has long been observed that elongated bacteria like *Legionella pneumophila* and *Borrelia burgdorferi*, hyphal fungi like *Candida albicans*, and parasites like *Leishmania* and *Trypanosoma cruzi* are engulfed by so-called “coiling phagocytosis,” in which phagocyte membrane protrusions wrap around the complex morphology of these microbes (212–214). In many cases the molecular mechanisms have only been partially explored. Yet, commonalities with the concepts described in this review are evident from the role of receptor binding and the formation of actin-based protrusions, which involve Arp2/3 and formin activation and signaling similar to those described for lamellipodia and filopodia formation (215). However, uptake of elongated or spiral shaped microbes or synthetic particles is generally less effective than that of spherical particles, and several biophysical models have suggested increased mechanical constraints linked to the uptake of complex shapes (7, 9, 28). For instance, prolate spheres or rod particles, a very common shape for microbes, can bind to phagocytes efficiently, but their internalization is markedly reduced compared to that of spherical particles (216). Remarkably, elongated particles are internalized more efficiently when their initial contact with the phagocyte occurs at the pole rather than the side (7). This phenomenon can be recapitulated in a two-stage model that combine passive diffusion-based receptor binding followed by a stage that actively promotes further receptor engagement (28). Thus, even for complex shapes, the same scheme of receptor binding, actin-based protrusion and coupling between the actin and particle-engaged receptors seems to apply. However, the mechanical burden associated with the formation of more geometrically complex phagocytic cups can become unsurmountable for the phagocyte. Consequently, elongated microbes can at least partially escape killing by phagocytosis thanks to their morphology, as observed for *Candida albicans* (217).

The diversity of phagocytic targets is managed by the expression by phagocytes of a plethora of different phagocytic receptors, which are able to recognize various opsonins, pathogen-associated molecular patterns and “eat me signals” (27, 218). While the engagement of these receptors likely involves similar concepts to those described here for FcγR and αMβ2, such as receptor lateral diffusion, formation of signaling clusters and activation of actin polymerization, specific properties of these receptors could vary greatly, and in most cases, remain largely under-characterized. For instance, the lateral diffusion of the scavenger receptor CD36 appears to be restricted by the cortical actin cytoskeleton, but displays anisotropic trajectories very distinct from those observed for FcγR or β2 integrins (41, 55, 219). Also, CD45 can inhibit signaling by the C-type lectin receptor Dectin-1, and appears to be excluded from the Dectin-1-mediated phagocytic cup (220). This is consistent with the presumed small size of Dectin-1 and the kinetic segregation model established for immunoreceptors. However, it is clear that the dimensions of phagocytic receptors vary dramatically, suggesting that some phagocytic events are likely to occur independent of a size-based segregation mechanism to regulate signaling (221). Thus, it is likely that the physical constraints and concepts presented here are broadly shared across the different phagocytic pathways, yet the details of their implications could vary and should be examined specifically for each individual case.

Finally, while this review is focused on the mechanics of internalization mechanisms, killing, processing and disposing of internalized material can represent tremendous constraints and limit phagocytosis capacity. In particular, cell turnover, and tissue homeostasis probably represent the largest burden on phagocytes, as it has been estimated that in humans, 200–300 billion cells are replaced every day (222). Given the scale of this task, it is not surprising that phagocytosis of dead cells, also called efferocytosis, must be shared between many cells, including professional and non-professional phagocytes, such as Sertoli cells and retinal pigmented epithelial cells. Moreover, in addition to the membrane surface area initially available on the phagocyte, the phagocytic capacity over time can be limited by the rate of degradation of the internalized material, which involves activation of appropriate enzymatic and metabolic pathways (223). It is therefore conceivable that phagocytes gather information regarding the physical properties of the ingested material, including their size and stiffness, in order to regulate their processing programs (25). How sensing of physical and molecular cues is integrated to regulate the broad range of phagocyte functions remains largely unknown and will be an exciting problem for the coming years.

## Author Contributions

VJ and CW wrote the manuscript. VJ prepared the figures.

## Conflict of Interest

The authors declare that the research was conducted in the absence of any commercial or financial relationships that could be construed as a potential conflict of interest.
